# The Effect of *Frankia* and *Hebeloma crustiliniforme* on *Alnus alnobetula* subsp. *Crispa* Growing in Saline Soil

**DOI:** 10.3390/plants11141860

**Published:** 2022-07-16

**Authors:** Haoran Chen, Sylvie Renault, John Markham

**Affiliations:** Department of Biological Sciences, University of Manitoba, Winnipeg, MB R3T 2N2, Canada; chenh318@myumanitoba.ca

**Keywords:** nitrogen fixation, actinorhizal plants, specific nodule activity, salt stress

## Abstract

The mining of the oil sands region of Canada’s boreal forest creates disturbed land with elevated levels of salts. Understanding how native plants respond to salt stress is critical in reclaiming these lands. The native species, *Alnus alnobetula* subsp. *crispa* forms nitrogen-fixing nodules with *Frankia*, and ectomycorrhizae with a number of fungal species. These relationships may make the plant particularly well suited for restoring disturbed land. We inoculated *A. alnobetula* subsp. *crispa* with *Frankia* and *Hebeloma crustiliniforme* and exposed the plants to 0, 50, or 100 mM NaCl for seven weeks. *Frankia*-inoculated plants had increased biomass regardless of salt exposure, even though salt exposure reduced nitrogen fixation and reduced the efficiency of nitrogen-fixing nodules. The nitrogen-fixing symbiosis also decreased leaf stress and increased root phosphatase levels. This suggests that N-fixing plants not only have increased nitrogen nutrition but also have increased access to soil phosphorus. Mycorrhizae did not affect plant growth but did reduce nodule numbers and nodule efficiency. These results suggest that the nitrogen-fixing trait is more critical than mycorrhizae. While salt stress inhibits nitrogen-fixing symbiosis, plants still benefit from nitrogen fixation when exposed to salt.

## 1. Introduction

Salinity is one of the most globally important abiotic plant stressors. About 400 million hectares of land are salt-affected (http://www.fao.org/soils-portal/soil-management, accessed on 1 May 2022). In cultivated lands, salinity stress is commonly associated with irrigation [1]. In uncultivated land, salinity stress can also result from industrial activities. The extraction of bitumen from oil sands in the boreal region of western Canada results in the production of saline tailings material [2,3]. Approximately 70,000 ha of land have been disturbed due to oil sands development [4]. Revegetating areas with this tailings waste is a challenge, in part due to its salt levels. 

Salt acts as an osmotic stress, restricting plant uptake of water, and an ionic stress, interfering with plant ion balance [5]. Both of these stresses can affect whole plant performance, reducing the mass of roots and shoots, plant height, leaf expansion, and basic physiological functions [6]. Most research on salt stress in plants has been conducted on crop species in temperate or arid regions. We know much less about plant responses to salt stress in the boreal region. This is especially true of nitrogen-fixing species, which could play a vital role in restoring industrially disturbed land in boreal ecosystems [7]. 

Actinorhizal plants form nitrogen-fixing nodules in association with the actinomycete *Frankia*. Many actinorhizal plants grow in low fertility soil and play an important role as pioneer plants early in plant community development [8]. *Alnus* is one of the most widely distributed actinorhizal genera [9,10]. In areas with a low availability of inorganic nitrogen, *Alnus* species rely heavily on N fixation, which not only meets their N requirement but also increases the fertility of the soil [11]. *Alnus* species, therefore, have the potential to be used in the reclamation of disturbed areas [4,12,13]. Some *Alnus* species are tolerant of soil salinity [14]. However, although *Frankia* can tolerate high salinity levels in vitro, nitrogenase activity is inhibited both in vitro [15] and in plant nodules [7]. Therefore, the benefit of using *Alnus* species in the reclamation of saline soils needs to be investigated. 

Green alder (*Alnus alnobetula* subsp. *crispa* (Aiton) Raus) is a native woody plant widely distributed across the boreal region of North America. Research on its use for the revegetation of disturbed areas has mostly focused on its nitrogen-fixing ability [12]. One field study demonstrated that *Frankia*-inoculated green alder on oil sands tailings capped with soil substantially increased soil quality, reducing soil pH from 7.5 to 6.6, plant-available sodium content by 70%, and increasing soil organic matter content after two growing seasons [16]. Another study found that nodulated green alder biomass was greater than non-nodulated plants when grown in pure oil sands mixed with sand [4], confirming the effect of the nitrogen-fixing symbiosis on plant performance.

In addition to forming a nitrogen-fixing symbiosis, *Alnus* species can also be colonized by ectomycorrhizal fungi (ECMF) [9]. Plant association with ECMF can play an important role in increasing nutrient uptake via phosphatase and protease production [17,18]. In nitrogen-fixing plants such as *Alnus*, symbiosis with ECMF can increase both plant growth and nitrogen fixation [19]. ECMF are also known to alleviate different types of abiotic stress on plants, making them valuable tools in land restoration [20]. Markham [21] showed the dual symbiosis of *Alnus incana* subsp. *rugosa* with ECMF (*Paxillus involutus*) and *Frankia* increased plant mass when plants grew in a mixture of peat and gold mine tailings with elevated levels of heavy metals. However, the mechanism by which mycorrhizae benefit plants exposed to mine tailings remains unclear. Several reports have described the use of ECMF to reduce salt stress in plants. Nguyen et al. [22] found that NaCl-treated *Picea glauca* and *Picea mariana* had reduced shoot chloride levels when inoculated with *Hebeloma crustuliniforme* or *Laccaria bicolor.* On the other hand, these ECMF species did not reduce shoot chloride in *Pinus banksiana* under the same conditions. In salt-stressed aspen (*Populus tremuloides*), association with *H. crustuliniforme* resulted in higher plant biomass and root hydraulic conductance than in non-mycorrhizal seedlings [23]. It has been suggested that ECMF could play an important role in preventing Na^+^ and Cl^−^ translocation to plant tissues and could enhance nutrient uptake, making ECMF a good choice for the remediation of tailings contaminated soil [22,24].

The aim of this study was to determine the effect of both *Frankia* and ECMF on the performance of green alder under the saline conditions typical of oil sand industrial activity. We predicted that *Frankia* and ECMF inoculation would have a synergistic effect on plant growth by alleviating the nutrient deficiency associated with salt stress. 

## 2. Results

**Figure 1:**
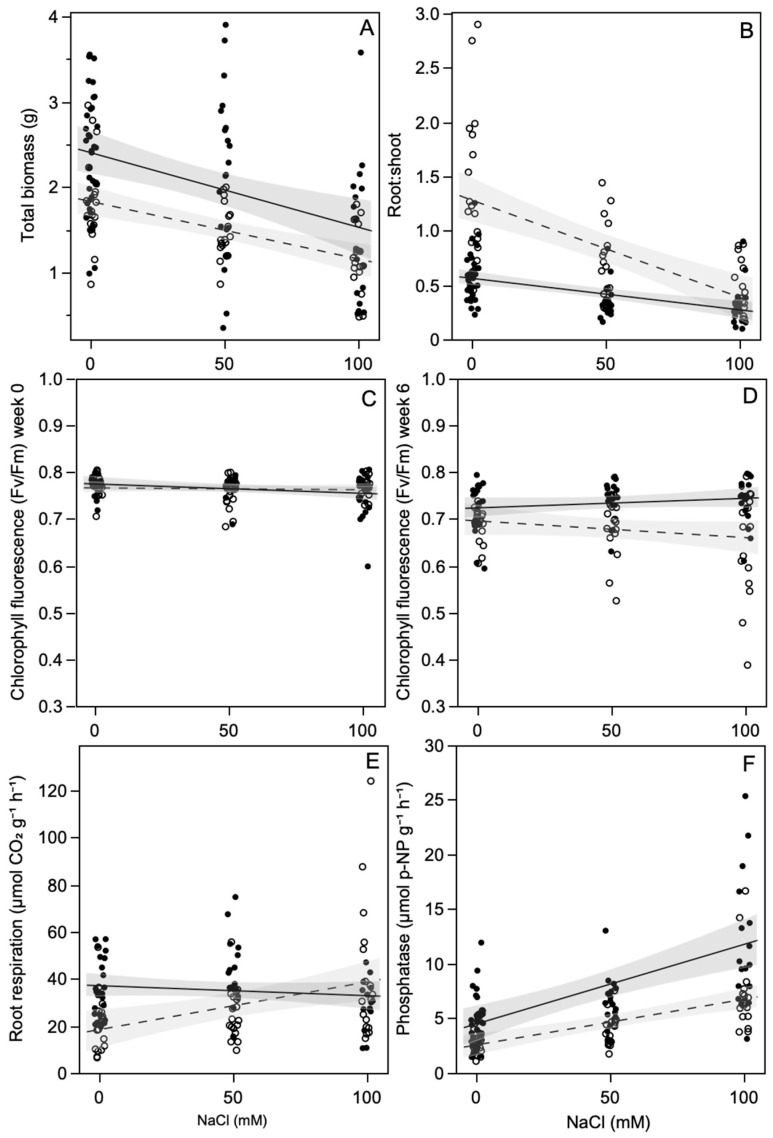
Effect of salt exposure on: (**A**) total plant mass, (**B**) root:shoot ratio, leaf chlorophyll fluorescence prior to (**C**) and six weeks after (**D**) salt exposure, (**E**) root respiration excluding nodules and, (**F**) root extracellular acid phosphatase activity, of *Frankia*-inoculated (closed symbols, solid line) and non-inoculated plants (open symbols, dashed line) averaged across ECMF inoculation treatments. Lines are least squares fits with 95% confidence intervals.

All but one of the ECMF-inoculated plants formed Hartig nets, and all but one of the *Frankia*-inoculated plants formed nodules visible to the naked eye. *Frankia* inoculation significantly increased total plant biomass by 37% compared with non-inoculated plants, regardless of mycorrhizal inoculation or salt level, i.e., there was no interaction between these treatments (Figure 1A, Table 1). Increasing NaCl significantly decreased total plant biomass, such that plants exposed to 100 mM NaCl were 40% smaller than plants exposed to no NaCl, regardless of *Frankia* or ECMF inoculation. Salt exposure had a stronger effect on the root:shoot ratio of non-*Frankia*-inoculated plants than *Frankia*-inoculated plants (Figure 1B). In the non-*Frankia*-inoculated plants, the root:shoot ratio was reduced by 66% in plants exposed to 100 mM NaCl compared to plants exposed to 0 mM NaCl. The *Frankia*-inoculated plants in the non-salt treatment had a lower root:shoot ratio than the non-inoculated plants, and it was reduced by 33% when the plants were exposed to 100 mM NaCl. Prior to the salt treatments, the chlorophyll fluorescence data showed that the plants were not stressed, and there was no difference between the treatments (Figure 1C). By week six, the maximum quantum yield decreased in all treatments, but the non-*Frankia*-inoculated plants had a lower maximum quantum yield, which decreased as the salt level increased. In the non-inoculated plants, root respiration (excluding nodules) increased by 42% for plants exposed to 100 mM NaCl compared to plants grown at 0 mM NaCl (Figure 1E). Salt exposure did not affect the respiration of *Frankia*-inoculated plants, and at 0 mM NaCl, the inoculated plants had a higher rate of root respiration than the non-inoculated plants. Root extracellular acid phosphatase activity increased with increasing salt exposure, with plants exposed to 100 mM NaCl having a 2.3 times higher level than plants not exposed to salt (Figure 1F). *Frankia* inoculation also increased root phosphatase by 1.5 times compared to non-inoculated plants, regardless of salt exposure. ECMF inoculation did not affect plant mass, root:shoot ratio, root respiration, or root phosphatase levels.

**Figure 2:**
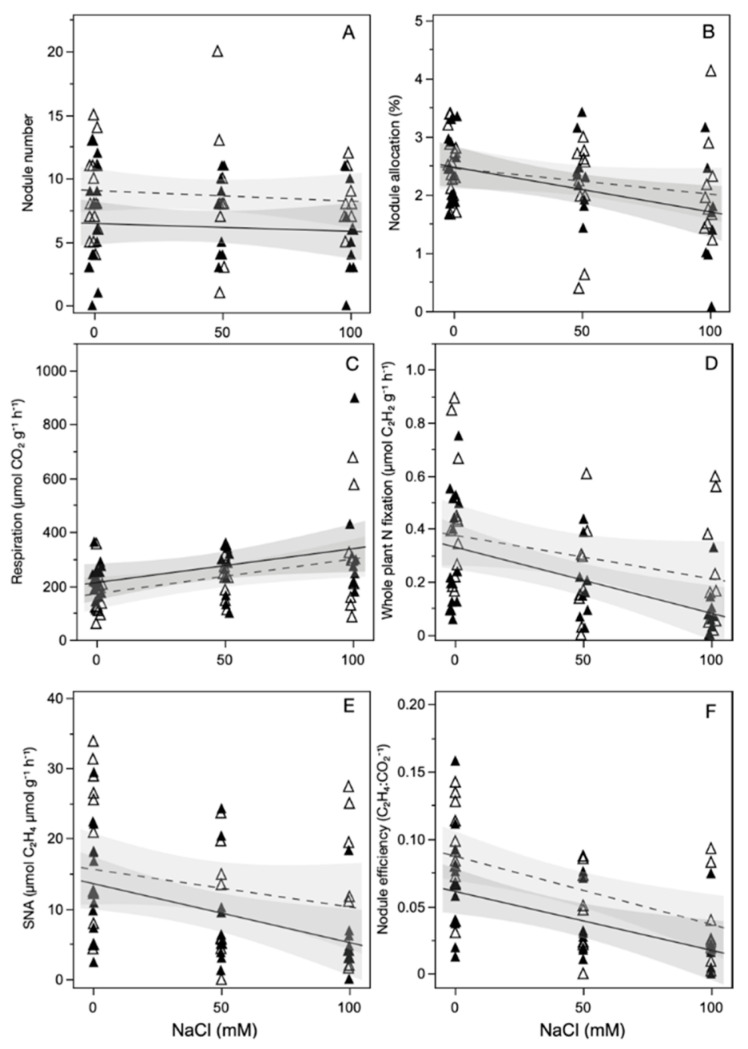
Effect of salt exposure on: (**A**) nodule number; (**B**) total plant biomass allocated to nodules; (**C**) nodule respiration; (**D**) nitrogenase activity relative to whole plant mass; (**E**) specific nodule activity; (**F**) the efficiency of nitrogenase activity, for plants that were inoculated with both Frankia and ECMF (closed symbols) or *Frankia* (open symbols).

Salt exposure did not affect the number of nodules on a plant at the time of harvest (Figure 2A, Table 2). However, salt exposure reduced all other measures of the nitrogen fixing benefits of *Frankia* inoculation. Compared to non-salt exposed plants, plants exposed to 100 mM NaCl had a 24% reduction in biomass allocation to nodules, a 64% increase in nodule respiration, and a 42% decrease in specific nodule activity. These measures combined reduced nodule efficiency by 56% and the amount of total nitrogenase activity per plant mass by 54% when plants were exposed to 100 mM NaCl. ECMF inoculation reduced nodule number by 28% and nodule efficiency by 33%, compared to non-inoculated plants, regardless of salt exposure level. 

## 3. Discussion

Our results show that the growth benefit of the nitrogen-fixing symbiosis in *A. alnobetula* subsp. *crispa* is not affected by salt exposure (i.e., there was no interaction between the *Frankia* inoculation and salt treatments on biomass. *Frankia* inoculation also reduced salt stress in leaves as indicated by the maintenance of the maximum quantum yield of photosynthesis in presence of salts. The increased growth of nitrogen-fixing plants occurred even though salt exposure decreased nitrogen fixation per plant mass and nodule mass and decreased the efficiency of nitrogen fixation in nodules. The decrease in nodule function reported here is consistent with our earlier study on salt exposure in *Eleagnus commutata* [7], in which we found a decrease in the formation of nitrogen-fixing vesicles in nodules of plants exposed to 100 mM NaCl. This decrease in vesicles could explain the reduced efficiency of nodules that we found in the current study. The lack of an effect of NaCl on nodule number in our plants was expected since plants were inoculated well before salt exposure, such that the plants would have had enough time to form nodules before experiencing any salt stress. Tailings from oil sand extraction have been shown to decrease nodule numbers in *A. alnobetula* subsp. *crispa* but not nodule allocation [4]. While nodulated plants benefit from a source of fixed N, they do not need to invest as much of their biomass in the production of roots for N absorption, allowing them to produce relatively more photosynthetic tissue [25]. The increase in root respiration resulting from salt exposure that we found has been observed in many species and has been attributed to the increased ion flux in roots [26]. This increased metabolic cost further reduces the ability of plants with a high root: shoot ratio to grow. Therefore, having N-fixing nodules not only provides plants with a source of N but also reduces root exposure to salt by decreasing the area of roots in contact with the soil. 

The higher acid phosphatase activity of *Frankia*-inoculated plants supports the prediction that N fixation can aid in P acquisition [27,28]. Phosphatase enzymes are N rich, and increased access to N can increase their production. Chodak and Niklinska [29] found soil under *Alnus glutinosa* had elevated acid phosphatase activity. Conifer-dominated stands with *Alnus rubra* were also shown to have increased soil P availability and plant tissue P [30]. However, increased phosphatase activity does not seem to be universal among N fixing plants. Png et al. [31] found that an actinorhizal plant (*Allocasuarina lehmannian*) did not increase phosphatase activity in response to decreasing soil P, but *Acacia* species did. They concluded that phosphatase activity in response to lower soil P was a phylogenetically constrained trait and not a result of having the nitrogen fixation trait. Our results show that even though salt stress reduced whole plant and nodule nitrogen fixation and the efficiency of nodules, nodulated plants increased root phosphatase activity. Similar to our results, previous studies have reported that salt and water stress can increase phosphatase activity in roots and shoots, presumably due to the reduced nutrient uptake associated with salt or water stress [32,33,34]. 

The lack of a mycorrhizal effect on plant performance was unexpected, given the low fertility of the soil used in this experiment. Studies on the effect of mycorrhizae on the performance of boreal forest plants under salt stress have been inconsistent. Muhsin and Zwiazek [35] found that white spruce in symbiosis with *H. crustuliniforme* and exposed to 25 mM NaCl had reduced accumulation of sodium in shoots and roots, increased N and P uptake in mycorrhizal roots, and increased ability for shoots to maintain high rates of transpiration under salt stress. Bois et al. [36] tested three ectomycorrhizal fungal species that were salt tolerant in vitro on greenhouse-grown conifers. The authors found that jack pine (*Pinus banksiana* L.) inoculated with *H. crustuliniforme* showed the greatest tolerance to salt stress (200 mM NaCl), while white spruce (*Picea glauca*) seedlings inoculated with *Suillus tomentosus* had the best growth even at 200 mM NaCl. However, Nguyen et al. [22] found little effect of *H. crustulimiforme* in altering the growth of *P. glauca*, *Picea mariana,* and *P. banksiana* when exposed to salt stress. Langenfeld-Heyser et al. [37] found no effect of mycorrhizal on the growth of hybrid poplar exposed to up to 500 mM NaCl. 

Similar to our findings, Ba et al. [38] found that mycorrhizae inhibited nodule formation in a legume species (*Acacia holosericea*). This likely results from ectomycorrhizae reducing root hair density, the site nitrogen-fixing bacterial infection. On the other hand, inoculating *Alnus tenufolia* with ECMF has been shown to increase nodule mass but not affect nitrogen fixation [19].

Our results suggest that *A. alnobetula* subsp. *crispa* is moderately salt tolerant, having a 40% reduction in growth at 100 mM NaCl. A number of actinorhizal plants have been found to have a high level of salt tolerance. Some *Casuarina* species are halophytes and can grow in 600 mM NaCl [39,40]. In *Casuarina equisetifolia,* nodule formation is not affected by NaCl up to a concentration of 150 mM [41]. *Shepherdia argentea* possesses high salt tolerance of up to 400 mM NaCl [42]. Some *Elaeagnus* species can persist in up to 300 mM NaCl [43,44]. *Elaeagnus* and *Shepherdia* have species found in boreal regions and warrant more study for their use in restoration. While it has been argued that N fixing plants could be particularly useful in restoring areas with saline soil, the reduction in N fixation in salt-stressed plants may compromise their utility. However, using N fixing plants may solve the problem of needing to supply nitrogen to the soil without causing nitrogen runoff. Our results also indicate that N fixing plants can potentially improve the P fertility of soils. 

## 4. Materials and Methods

*Alnus alnobetula* subsp. *crispa* seeds collected from the Sandilands Provincial Forest (Latitude: 49.3 N, Longitude: 96.1 W) were stratified at 4 °C for two weeks and then sown in sterilized peat moss and perlite (3:1, *v*/*v*) and grown under artificial light (T5 HP fluorescent lamps) with a 16-h light: 8-h dark photoperiod. The relative humidity (RH) ranged from 30% to 50%, and the temperature was 25 °C/20 °C (day/night). At four months, we transferred seedlings to 6.25 cm diameter × 25 cm deep pots (D40H Deepots, Stuewe & Sons, Tangent, OR, USA) filled with soil from the forest where we collected the seeds. The soil is 99.8% sand by mass and has an inorganic N content of 10.2 ± 0.6 mg kg^−1^ (mean ± standard deviation) and phosphate level of 0.98 ± 0.33 mg kg^−1^ [45]. The soil was autoclaved for one hour. Seedlings were inoculated with *Frankia* and/or the ECMF species, *Hebeloma crustiliniforme*, with control plants receiving no inoculation. This resulted in four invocation treatments: 1. no *Frankia*, no ECMF, 2. *Frankia*, no ECMF, 3. No *Frankia*, ECMF and, 4. *Frankia* and ECMF. The *Frankia* inoculant consisted of surface-sterilized field-collected crushed nodules at 2 mg mL^−1^ of PBS Buffer. Each plant was inoculated by injecting 2 mL of the inoculant on the surface of the soil. An *H.* *crustiliniforme* culture (UAMH 6064) was obtained from the University of Alberta Mycological Herbarium and was originally isolated from a sporocarp collected under *A. alnobetula* subsp. *crispa*. The fungal strains were maintained at 25 °C in the dark for over 2 months on plates containing Modified Melin Norkrans (MMN) agar medium (composition: glucose 2.5 g, malt extract 2.0 g, yeast extract 1.0 g, potassium phosphate monobasic (KH_2_PO_4_) 0.5 g, ammonium phosphate dibasic ((NH_4_)_2_HPO_4_) 0.25 g, magnesium sulphate (MgSO_4_) 0.15 g, calcium chloride (CaCl_2_) 0.05 g, sodium chloride (NaCl) 0.025 g, ferric chloride (FeCl_3_) 0.012 g, agar 15 g, per liter distilled water) agar plates [45]. For the ECMF inoculation, three 3.9 mm diameter by ca. 7 mm height MMN agar plugs with active growing hyphae were placed next to the upper roots of the plant. Six weeks after inoculating the plants, the salt treatments were started. The salt concentrations (0, 50 and 100 mM NaCl) selected correspond to the range of concentrations found in the areas impacted by the oil sand industry. Ten plants in each combination of inoculation treatments received one of three salt treatments: 1. no NaCl (25 mL water), 2. two 25 mL doses of 50 mM NaCl (146 mg per pot), and 3. four 25 mL doses of 50 mM NaCl (292 mg per pot), hereafter referred to as 0, 50, or 100 mM salt treatments, respectively. Salt doses were applied once daily until the appropriate salt level was reached. Plants were then watered with distilled water to about saturation every two days for the remainder of the experiment. Each pot had a small cup underneath to collect the leaching solution from the soil, which we poured back onto the soil surface. Plants were exposed to NaCl conditions for seven weeks and then harvested and freeze dried to determine their biomass. 

Chlorophyll fluorescence was measured using an Opti-Sciences OS30p chlorophyll fluorometer (Hudson, NH, USA) the week prior to and six weeks after applying the salt treatments. Leaves were dark acclimated for 30 min prior to taking measurements. For the data collected at six weeks, measurements were taken on each plant on two different days, and the values averaged. We used the maximum quantum yield (*fv*/*fm*) as an index of plant stress. At the time of harvest, roots with attached nodules were cleaned of soil with room temperature water. We measured whole root respiration (with nodules attached) by sealing the roots in a 50 mL cylinder, pumping air through the cylinder at 0.2 L min^−1,^ and then through a desiccant tube before measuring the CO_2_ concentration using a CO_2_ analyzer (Qubit S151 CO_2_ Analyzer, Kingston, ON, Canada). We then removed the nodules from the plant, and their respiration rate was measured using the same procedure. The difference in CO_2_ concentrations between the whole root system and nodules was used to calculate root respiration in the absence of nodules. Samples were then freeze dried, and respiration rates were expressed on a dry mass basis. 

To examine mycorrhizal formation, pieces of fresh roots were weighed and stored in 70% ethanol. The dry weight of these pieces was estimated using the dry to fresh weight ratio of the remaining roots. Mycorrhizae formation was confirmed by examining up to 10 sections of fine roots at 400× magnification for the presence of a Hartig net. Root sections were pre-selected for microscopic examination by the presence of brown flexuous fine roots or succulent fibrous root tips [46].

Acetylene reduction assays were performed on the nodules following [25]. Nodules were sealed in 50 mL containers with an atmosphere of 10% acetylene for one hour. Ethylene in the headspace was then measured using a gas chromatograph (Varian 4500, Edmonton, AB, Canada) with a gas sampling valve and a Haysep T column connected to an FID. The specific nodule activity (SNA) was expressed as the ethylene production rate per nodule dry mass. Nodule allocation was calculated as the nodule mass as a percentage of the total dry mass. We measured root acid phosphatase activity according to [47]. Briefly, root samples without nodules were incubated in the dark for one hour with 3.5 mM para-nitrophenyl phosphate (p-NPP) with a 100 mM modified universal buffer. The reaction was then stopped with 1M NaOH, and the absorbance of the filtered solution at 410 nm compared to a standard curve of para-nitrophenyl (p-NP). The phosphatase enzyme activity was expressed as µmol p-NP per hour per gram of dried root. 

The data were analyzed using a three-factor least squares model with *Frankia* and ECMF inoculation as nominal effects and NaCl level as a continuous effect, with ten replicates per treatment. The nodule number per plant data was analyzed using a GLM model with a Poisson distribution. When there was a significant interaction between NaCl and the other factors, the effect of salt on the response variable was analyzed using linear regressions for each level of the interacting factor.

## Figures and Tables

**Table 1:** *p* values from least squares models of the effect of *Frankia*, ECMF, and salt exposure on plant performance.

	Total Mass	Root: Shoot	Extracellular Phosphatase *	Root Respiration	Chlorophyll Fluorescence	
					Week 0	Week 6
Frankia	<0.0001	<0.0001	<0.0001	0.0142	0.9729	<0.0001
ECMF	0.5392	0.5599	0.3161	0.6995	0.2407	0.1390
NaCl	<0.0001	<0.0001	<0.0001	0.0258	0.0808	0.7787
Frankia × ECMF	0.5384	0.7750	0.6225	0.6394	0.4793	0.3789
Frankia × NaCl	0.4615	0.0001	0.7563	0.0017	0.2561	0.0268
ECMF × NaCl	0.9535	0.6063	0.6772	0.3803	0.1492	0.0603
Frankia × ECMF × NaCl	0.1458	0.2164	0.1330	0.3955	0.2882	0.0522

* Analysis performed on log transformed data.

**Table 2:** *p* values from least squares models of the effect of ECMF and salt exposure on nodule number, biomass allocation to nodules, nodule respiration, specific nodule activity (SNA), and nodule efficiency.

	Nodule Number	Nodule Allocation	Nodule Respiration	SNA	Nodule Efficiency
ECMF	<0.0001	0.5198	0.2023	0.1389	0.0100
NaCl	0.3617	0.0065	0.0015	0.0122	<0.00001
ECMF × NaCl	0.9911	0.4512	0.9695	0.5681	0.7450

## Data Availability

Data are available from the corresponding author.
